# The Efficacy of Targeted Mindfulness-Based Interventions for Improving Mental Health and Cognition Among Youth and Adults with ACE Histories: A Systematic Mixed Studies Review

**DOI:** 10.1007/s40653-022-00454-5

**Published:** 2022-05-05

**Authors:** Ellie Moyes, George Nutman, Jessica Hafetz Mirman

**Affiliations:** Department of Clinical and Health Psychology, School of Health in Social Science, University of Edinburgh, Edinburgh, Scotland

**Keywords:** Targeted intervention, Mindfulness-based intervention, Mindfulness-based intervention, Adverse childhood experience, ACE, Adversity

## Abstract

Mindfulness-based interventions (MBIs) are associated with increased psychological wellbeing. The literature suggests that individuals exposed to adverse childhood experiences (ACEs) may benefit greatly from MBIs. However, research has tended to focus on universal MBIs for this population with less attention on the effectiveness of targeted approaches. Moreover, there is growing concern regarding the methodological rigor of MBI research. This systematic mixed studies review (SMRS) reports the effectiveness of MBIs for improving mental health and cognition among individuals with ACE histories. Additionally, the review reports the quality and rigor of the included research. Systematic searches of PsycInfo, EMBASE, MEDLINE, ProQuest Dissertations and Theses, ProQuest Social Science database and the Child Development and Adolescent Studies database were conducted. Results were screened and data were extracted then synthesized using a data-based convergent synthesis design. Thirteen studies were included in the final review. Six prominent themes emerged. Themes indicated that MBIs were effective for improving mental health and cognition for individuals with ACEs. For example, improvements in mood and anxieties, as well as a better ability to manage emotions. Shortcomings in the quality of MBI research included lack of reporting of methodological details (e.g., randomization procedures) and not systematically reporting adverse event evaluations. Recommendations are made for future research to strengthen the evidence base for MBIs for individuals with ACEs.

**Supplementary Information:**

The online version contains supplementary material available at 10.1007/s40653-022-00454-5.

Mindfulness is the state of being fully aware in the present moment and being non-judgmental towards oneself (Kabat-Zinn, 1990), with higher levels of mindfulness being associated with greater psychological well-being (Bränström et al., 2011; Schutte & Malouff, 2011) and less psychological stress (Hicks et al., 2020). It has been conceptualized as both a trait, i.e. an innate characteristic, and a state, i.e. a skill that can be practiced (Rau & Williams, 2016; Tang, 2017). Research suggests that practicing mindfulness improves state mindfulness and overtime can increase trait mindfulness (Kiken et al., 2015). Therefore, it is no surprise that recent years have seen the emergence of mindfulness-based interventions (MBIs) that aim to harness the therapeutic benefits of mindfulness practices (Klingbeil et al., 2017).

## MBIs

Several standardized MBIs exist (Chiesa & Malinowski, 2011) such as: mindfulness-based stress reduction (MBSR; Kabat-Zinn, 1990), mindfulness-based cognitive therapy (MBCT; Segal et al., 2002), acceptance and commitment therapy (ACT; Hayes et al., 1999), dialectical behaviour therapy (DBT; Linehan, 1993), and Mindful Self-Compassion (Germer & Neff, 2019). Mindfulness techniques are also incorporated into several unstandardized interventions, e.g., yoga, art therapies and other mind–body interventions (Breedvelt et al., 2019; Ortiz & Sibinga, 2017; Varambally & Gangadhar, 2016). All MBIs aim to teach individuals to recognize and accept unpleasant thoughts and feelings; to reflect on their reaction to these; and to apply appropriate coping skills (Gu et al., 2015; Shapero et al., 2018).

### Efficacy of MBIs

There is a well- established evidence base supporting the use of standardized MBIs in treating mood and anxiety disorders (Lynch et al., 2003; Maiello et al., 2020; Segal & Teasdale, 2018), and borderline personality disorders (O’connell & Dowling, 2014). So much so that the National Institute for Care Excellence endorses MBCT as an effective treatment for preventing relapse in clinical depression (Crane & Kuyken, 2013). Reviews also report the benefits of yoga and other mind–body interventions in improving depression and increasing mental health generally (Bridges & Sharma, 2017; Cramer et al., 2013; Domingues, 2018), particularly when delivered in adjunct with other treatments (Taylor et al., 2020). Preliminary research also supports the use of MBIs for bi-polar disorders and eating disorders (Dunne, 2018; Key et al., 2017; Salcedo et al., 2016). Moreover, MBIs are reported to be more effective than placebo or treatment as usual for most psychiatric disorders, more clinically effective than psychoeducation and support groups, and comparable to traditional cognitive behaviour therapy (A-Tajk et al., 2015; Goldberg et al., 2018; Ruiz, 2012). The dissemination of MBIs, particularly non-standardized approaches, in non- clinical settings (Baer et al., 2019) has highlighted the positive effects of MBIs on non-pathological indicators of wellbeing, including cognition (Felver et al., 2016). Therefore, MBIs may be of greater benefit to vulnerable populations and individuals (Baer et al., 2019), such as those who endure adverse childhood experiences (ACEs) (Ortiz & Sibinga, 2017; Felitti et al., 1998).

### ACEs and Developmental Outcomes

There are ten common ACEs – neglect; physical, sexual or emotional abuse; exposure to violence, mental illness, incarceration, or substance abuse in the family; and parental absence due to divorce or separation (Felitti et al., 1998) – with low socioeconomic status, community violence and being removed from the family home now also being recognized (The Scottish Government, 2012). Exposure to ACEs is attributable to the onset of approximately one-third of all mental disorders (Green et al., 2010; McLaughlin et al., 2012). For example, exposure to violence in childhood contributes to attentional biases toward threat cues (Lambert et al., 2017), that confer a risk factor for anxiety and PTSD (Shackman et al., 2007). Early life adversity is also associated with deficits in executive functioning (Nusslock & Miller, 2016). Specifically, childhood exposure to poverty (Javanbakht et al., 2015) and neglect (Maheu et al., 2010) are common risk factors for heightened emotional reactivity and an increased use of maladaptive emotion regulation strategies (Heleniak et al., 2016, 2018), which confer risk of depression, anxiety and PTSD (Chapman et al., 2004; Gratz et al., 2008; McElroy & Hevey, 2014).

### MBIs for Individuals with ACE Histories

For individuals with ACE histories, MBIs may work to improve use of coping strategies and overall mental health by recognizing and managing the negative thoughts and emotions that are common outcomes of ACE exposure (Baer et al., 2019; Sheffler et al., 2020). The majority of MBI research has used universal strategies to reach the target population at mass through institutions where the population is commonly found (Dodge, 2020; Sanders & Morawska, 2011). Although results do indicate improvements in mental health and cognition for ACE individuals of all ages through universal MBI approaches (McKeering & Hwang, 2019; Simpson et al., 2018), a potential issue is that there is no real certainty regarding the extent to which the target population will be reached (Greenberg & Abenavoli, 2017). For this reason, targeted strategies that specifically aim to target sub-groups/individuals within the target population (Horowitz & Garber, 2006) are perhaps preferable (Dodge, 2020). Research has found targeted MBIs to be useful in teaching adult survivors of ACEs how to accept and explore their thoughts and feelings related to prior adversity (Follette et al., 2006; Gallegos et al., 2015; Kalmanowitz & Ho, 2016; Kimbrough et al., 2010). Importantly, results are found to maintain over time (Earley et al., 2014). Research using adult cohorts is much more prevalent than children and adolescents (Kirlic et al., 2020). It could be argued that there should in fact be greater focus on the latter population; as there is reason to believe that childhood and/or adolescence may be the optimal time to implement MBIs (Dunning et al., 2019). This is mainly because brain plasticity is greatest during this period and so children/adolescents may find learning and retaining mindfulness skills easier than adults (Belsky, 1997; Blakemore & Choudhury, 2006).

### Concerns in MBI Research

Despite increases in research and dissemination, critics often note methodological shortcomings in MBI research (Gu et al., 2015): failure to utilise rigorous randomization processes (Goyal et al., 2014); variability in intervention style (Shonin et al., 2013); and concern regarding the potential for participants to experience adverse effects when undertaking MBIs, such as re-experiencing traumatic memories (Brewin, 2015; Lomas et al., 2015; Van Dam et al., 2018). Thus, the purpose of this review is to address the gap in the literature concerning the effectiveness of MBIs among individuals with ACE histories, specifically taking into account methodological rigor. Uniquely, we included studies with young people and adults thus enabling us to ascertain if there is support for the use of MBIs as both an early intervention, to foster positive changes for youth who experience adversity, and as a late intervention, for adults living with the persistent impact of ACE histories.

### Research Questions:

1. Are targeted MBIs effective for improving mental health and cognition among individuals with ACE histories?
2. What is the methodological quality and rigor of research pertaining to targeted MBIs for individuals with ACE histories?

## Methods

A systematic mixed studies review (SMSR; Pluye & Hong, 2014) was conducted. Conducting an SMSR is a highly interpretative protocol (Petticrew et al., 2013) that is best-suited for reviews that aim to synthesize data from studies that vary in methodology (e.g., quantitative, qualitative), that consider more than one type of outcome or research question, and that are interested in illuminating issues related to intervention reception (see Harden, 2010). This approach can facilitate knowledge synthesis by providing processes for considering methodologically distinct studies to contribute data to the same evidence (literature) analysis. In addition to standard systematic review procedures, SMSRs go a step further to coherently synthesize the findings across methods. Two independent researchers were utilized throughout the searching, screening, data extraction and quality analysis procedures to reduce risk of bias. Discrepancies at any stage were discussed and reconciled by the research team. The research protocol was informed by PRISMA guidelines (Moher et al., 2009). The authors have no conflicts of interest to declare in relation to this manuscript.

### Search Strategy

The literature was searched from 1st January 2010 to 10 August 2021 using PsycInfo, EMBASE, MEDLINE, ProQuest Dissertations and Theses, ProQuest Social Science database and the Child Development and Adolescent Studies database. The search terms ‘mindful* OR MBCT OR MBSR’; ‘child* OR adolescen* OR youth* OR young OR adult*’; ‘advers* OR ACE* OR “adverse childhood experience*” OR trauma*’ were combined with the ‘AND’ Boolean operator. The truncation (*) was included to increase the sensitivity of search terms.

### Eligibility Criteria

Eligibility criteria was informed by the PICOS (population, intervention, comparison, outcome, study design) method (Methley et al., 2014). Studies were included if they met all of the following criteria: (1) Participants had ACE histories; (2) The MBI was implemented in a targeted manner or mindfulness was the theoretical basis for the targeted intervention; (3) Comparison was either within or between subjects; (4) Outcomes measured mental health and/or cognition; (5) Studies were primary sources of literature; (6) Were published in English; and (7) Were published from 1st January 2010 onwards. No age restrictions were used.

### Screening Procedure

**Fig. 1:**
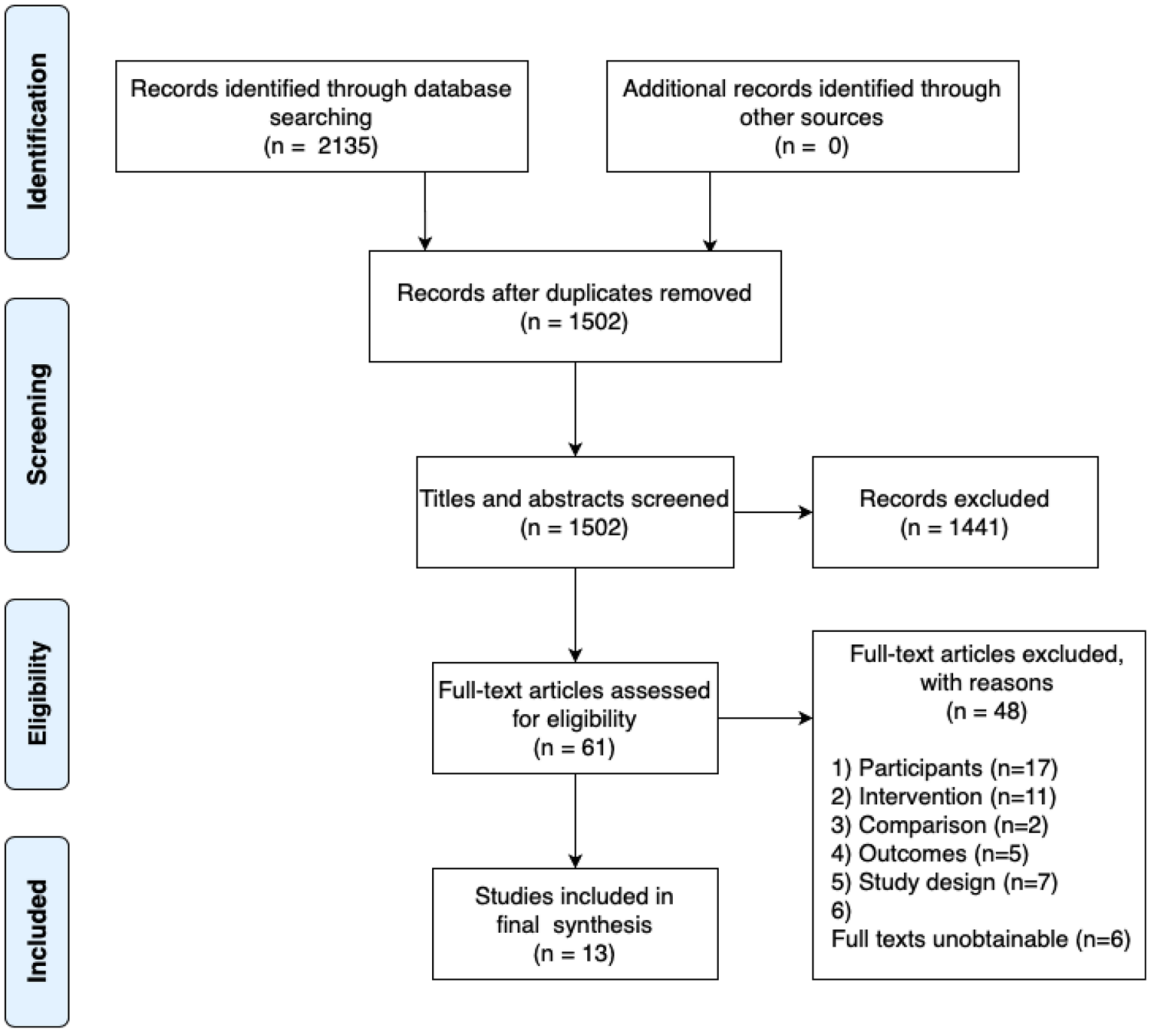
Flowchart of PRISMA screening procedure

The screening process (Fig. 1) was informed by PRISMA guidelines (Moher et al., 2009). Interrater reliability was calculated to be, κ = 0.62 (Cohen, 1960). The search yielded 1502 (de-duplicated) results, 55 were screened against inclusion criteria and review aims; of these, 13 were included in the final review.

### Data Extraction and Synthesis

A data-based convergent synthesis design was used (Hong et al., 2017). Firstly, both quantitative and qualitative data were extracted. Quantitative data were then transformed into qualitative categories by grouping outcome data in terms of domain being measured. This resulted in eight categories (mood, anxiety and stress, emotion, coping, social functioning, behaviour and cognitive functioning, psychological irritability and self-acceptance). A thematic analysis of all qualitative data was then conducted. Data extraction was conducted independently by two researchers prior to quality analysis. This approach reduces bias by blinding researchers to study quality (Boland et al., 2017).

### Quality Analysis

Methodological quality of the included studies was assessed using the Mixed Methods Appraisal Tool (MMAT) (Hong et al., 2018a). It provides five criteria points for appraising different types of methodology. Ratings of ‘*yes’* (clearly described by author), ‘*no’* (not mentioned by author or not met) or ‘can’t tell’ (unclear description given by author) can be given (Hong et al., 2018a).

## Results

Study characteristics, demographics, and outcome assessments are shown in Table 1. Eleven studies quantitatively measured outcomes. Across these, improvements in mental health and cognition were reported for individuals with ACEs. Improvements were found across most measures, although variability in statistical significance was noted; trauma symptoms did not improve. Quantitative data were grouped into eight broad domains of mental health and cognition. These were mood (1; 2; 6; 11; 13), anxiety (1; 6; 8; 10; 13), emotion (4; 5; 11), coping (1; 4; 5; 6; 7), social functioning (4; 8; 10; 13), behavior and cognitive functioning (4; 5; 7), psychological inflexibility (3; 8; 10; 13) and self-awareness (3; 13). Improvements were evident across these domains, regardless of MBI type or sample age.

**Table 1:** Full descriptions of study, sample and interventions characteristics

***Study*** ***ID***	***Author***	***Sample*** ***(N, age range, mean, standard deviation, gender)***	***Study design***	***ACE***	***Intervention***	***Main Findings***
1	Kimbrough et al. (2010)	N = 27 23-68y (M = 45, SD = 10.8) 24F, 3 M	Exploratory mixed- methods (within- subjects)	Childhood sexual abuse	Modified MBSR	A reduction in depression, anxiety & PTSD scores that remained at follow up An improvement in mindfulness scores that remained, marginally, at follow up
2	DePrince and Shirk (2013)	N = 2 15y F	Quantitative case study (within- subjects)	Parental domestic violence & financial difficulties	Adolescent Mood Project (adapted)	Reduction in depression symptomology throughout and immediately post-treatment; this maintained for 1 participant
3	Burrows (2013)	N = 1 18y F	Quantitative case study (within- subjects)	Sexual assault, parental substance use, parental mental illness, parental domestic violence & paternal incarceration	ACT	Moderate reduction in experiential avoidance and increase in psychological flexibility A reduction in thought suppression Notable reduction in trauma symptomology across the course of the intervention – although this was not a goal of the study
4	Sitzer and Stockwell (2015)	N = 43 9-12y 24F, 19 M	Quantitative descriptive (within- subjects)	Witnessing domestic violence	The Wellness Programme	Pre/post intervention differences in overall functioning were significant (0.777, p < 0.001) All 5 measures (behavioral, cognitive, emotional, social & resilience factors) yielded statistically significant differences pre/post-intervention; three of which at the 0.05 level or better
5	Caldwell and Shaver (2015)	N = 39 21-80y (M = 47, SD = 13.5) F	Quantitative quasi- experimental (between- groups)	Childhood maltreatment	REAC^2^H	Treatment group showed significant improvements in most of the study’s outcome variables, compared to control group
6	Jee et al. (2015)	N = 42 14-21y (M = 16.8, SD = 1.8) 23F, 19 M	Mixed- methods RCT	Removed from parents & family home	MBSR	No significant difference between pre/post intervention for general mental health, mindfulness and state/trait anxiety N.b. Sub-group analysis found a trend towards improvement on these measures for 14–17-year olds
7	Houser (2015)	N = 34 F [Site 1] (M = 13.68) [Site 2] (M = 15.89)	Embedded mixed methods (within- subjects)	Complex childhood trauma (emotional/ physical/ sexual abuse; neglect; or exposure to domestic violence)	Hatha yoga	Decrease in overall mental health scores; this was significant for site 1, but not site 2 Strong negative correlation between affirmation and mental health functioning, *r*(24) = -0.697, *p* < 0.001, and CPSS scores, *r*(11) = -0.661, *p* = 0.014 Intervention length had a significant effect on overall mental health symptom severity, F(1,9) = 4.66, p = 0.059, pἠ2 = 0.34. A moderate effect size was found
8	Spidel et al. (2018)	n = 50 19-64y (M = 40.4) 26F, 24 M	RCT	Childhood trauma	ACT	Intervention group showed greater improvements on areas such as psychiatric symptom severity, anxiety symptoms and the acceptance domain of emotional regulation; and showed increased help-seeking in the service engagement domain at 3 months follow up compared to baseline, relative to control (TAU) group
9	Norman (2018)	n = 8 12-18y M	Embedded mixed methods (within- subjects)	Maternal death, physical/sexual abuse, removed from family home & “unspecified psychological abuse”	The Mindfulness Curriculum for Adolescents	Clients who practiced mindfulness techniques regularly reported increasingly feeling calm and in control, an increased sense of relaxation and reduced aggression
10	Spidel et al. (2019)	n = 50 19-64y (M = 40.4) 26F, 24 M	RCT	Childhood maltreatment	ACT	No significant 3-way Time x Group x CTQ (childhood trauma questionnaire) score interaction across emotion, psychiatric symptoms, anxiety and help-seeking scores A *k*-means cluster analyses found three outcome clusters for the intervention group; a chi-squared analyses and Cramer’s V effect sizes indicated that none of the CTQ subscales’ were different across the clusters Increased attendance in ACT sessions, and an avoidant attachment style, were associated with cluster membership such that being in the two clusters that showed most improvements in clinical symptoms, increased help- seeking and acceptance
11	Van der Gucht et al. (2019)	n = 13 13-18y (M = 15, SD = 1.15) 5F, 8 M	Exploratory mixed methods (within- subjects)	Removed from parents, community violence/conflict, deprivation & financial difficulties	MBSR/MBCT: elements of each adjusted to suit population	Changes across mental health domains pre/post interventions of varying significance Depression symptoms positively correlated with negative affect Girls indicated higher correlation between depression symptoms, than boys Age was uncorrelated with outcomes
12	Fields (2019)	n = 5 21-31y F	Embodied qualitative (within- subjects)	Childhood sexual abuse	TIY	After 18 week intervention there were notable reductions in psychological/physiological symptoms, increased self-acceptance/self-awareness (mindfulness), body reconnection and improved relationship/affect regulation
13	Classen et al. (2020)	n = 32 24-64y F	RCT	Complex childhood trauma	TBG	Intervention significantly improved anxiety, body awareness and soothing receptivity – maintained at 6 month follow up Intervention had no significant impact on depression or PSTD

The quantitative results and the qualitative results from 6 studies were thematically analyzed resulting in six main themes (T):

(*T1) Improvements in mood.* This was mainly devised of improvements in depression symptoms although improvements in negative emotion also contributed (1; 2; 6; 7; 8; 10; 11; 13).

*(T2) Improvements in anxiety and/or stress.* Improvements in anxiety and increased calmness and/or relaxation and less rumination all account for this theme (1; 2; 3; 5; 6; 7; 8; 1 0; 13).

*(T3) Increased psychological flexibility.* This is the ability to adapt in daily life. This theme was established through increased acceptance of one’s thought and feelings, a better understanding of one’s self, increased body awareness and valued living (3; 4; 7; 11; 13).

*(T4) Increased understanding and management of emotions.* Across studies a better understanding of feelings and emotions, and how to manage these, was found (5; 4; 11).

*(T5) Increase in social skills.* A novel finding was that participating in MBIs increased participants social functioning. Specifically, by eliciting feelings of acceptance and being more comfortable to seek support (4; 8; 10; 13).

*(T6) Increased use of effective coping strategies.* Findings consistently suggested that participation in MBIs improved the use of effective coping strategies to cope with the effects of trauma, namely, mindfulness (1; 5; 6; 4; 7; 13).

### Study Quality

Only 3 studies met all five criteria points outlined by the MMAT (Hong et al., 2018a, b), seven studies met four criteria points and two studies met three criteria points. One study (6) was a mixed-methods RCT and was appraised using both quantitative RCT and mixed-methods study design criteria. The most commonly unmet criterion was that differences and inconsistencies between quantitative and qualitative results were not adequately addressed; this occurred in four mixed-methods study designs (1; 6; 7; 9). Randomization was not appropriately performed in two RCTs (8; 13), I.e. there was a lack of description of the randomization process (Hong et al., 2018a). It was unclear if participants were representative of the target population for two quantitative non-randomised trials (2; 3). This was because these were both case studies using n = 1 (3) and n = 2 (1) participants. Finally, in one non-randomized quantitative study (3) the intervention was not administered as intended; this was because the MBI ended abruptly due to the participant gaining employment and being unable to gradually end the intervention as planned for a full description of study alignment against quality criteria).

Five studies (1; 5; 6; 11; 13;) reported dropouts between originally recruited and final reported samples. However, baseline characteristics of dropped out participants were reported to not significantly differ from other participants. Thus, original authors did not deem analysis to be influenced by attrition. Follow-up data was available in five studies (1; 2; 3; 8; 13). Follow-ups ranged from four weeks to six months. Across studies follow-up data showed changes in mental health and cognition to maintain. Burrows et al. (3) noted maintained improvements in Client A, but not Client M; however, author notes this may have been due to personal adversities Client M faced since intervention ended. Effect sizes were reported for six of 13 studies (1; 7; 8; 10; 11; 13). One study explicitly mentioned study related adverse effects (1).

## Discussion

In this systematic review we evaluated the state of the evidence to determine if targeted MBIs are effective for improving mental health and cognition among individuals with ACE histories. Our results have implications for practice, theory, and future research and we consider these each in turn.

### Implications for Practitioners

From a programmatic and intervention standpoint, the main findings of our review indicate that MBIs are effective for improving mental health and cognition (Domingues, 2018; Dunning et al., 2019; Maiello et al., 2020). Moreover, this SMSR has highlighted the effectiveness of using targeted MBIs to improve these domains among individuals with ACEs histories (Baer et al., 2019; Ortiz & Sibinga, 2017; Sheffler et al., 2020). MBIs deployed in practice in the context of care should ideally be accompanied by a detailed implementation strategy, service utilization plan, quality assurance monitoring and an impact evaluation once the program is established. Finding resources for evaluation and quality monitoring on top of those needed for implementation can be challenging. University-community partnerships are one way to help to alleviate some of the resource constraints related to evaluation support where an exchange of expertise, knowledge, training opportunities (e.g., clinical intervention delivery, data analysis) between partners can potentially be leveraged to support evaluation and quality improvement activities.

### Implications for Theory

By utilizing a data-based convergent synthesis approach (Hong et al., 2017) this review provides a unique preliminary understanding of the processes that may underpin these improvements. These can perhaps best be understood in relation to the aims of MBIs – acceptance of, reflection on and ability to coping with thoughts and feelings (Gu et al., 2015; Shapero et al., 2018; Hayes et al., 2011; Seligman et al., 2014). For example, improvements in psychological flexibility were indicated (T3). Specifically, improvements in self-understanding, increased self-acceptance and an increased value of living. Overall, these factors denote an increased ability to accept one’s thoughts and feelings. For individuals with ACEs this may include the acceptance of suppressed thoughts related to the adversities experienced (Follette et al., 2006). The paradoxical effect whereby individuals who suppress negative thoughts actually ruminate over these more, results in greater distress (Wang et al., 2020). Therefore, the current findings suggest that by participating in targeted MBIs individuals with ACE histories may come to accept such negative thoughts and, in turn, improve overall psychological wellbeing (Ford et al., 2018).

Moreover, two themes indicated a better understanding and management of anxiety and/or stress (T2) and emotions (T4). This suggests that targeted MBIs improved participants’ ability to reflect on the aforementioned thoughts and feelings. Such acceptance and reflection of one’s thought and emotions equips individuals with a better ability to rationalize and utilise effective coping strategies (T6) (Aldao & Plate, 2018). Increased coping is also associated with improvements in mood (Arlt Mutch et al., 2020). This was also implicated to be an improved outcome by the present review (T1). This suggests that the themes derived from the current analysis have interlinking factors and that targeted MBIs may be efficacious across multiple domains concurrently. Although these aims are not solely designed for individuals with ACEs, the findings nonetheless begin to substantiate the use of targeted MBIs with this population.

Interestingly, the analyses in this review noted the emergence of a theme that associated targeted MBIs with increased social skills (T5). Syntheses suggested that these feeling often derived from participants enjoying being part of a group of people with similar experiences. This rich qualitative data imposes an argument that group variations of targeted MBIs may be more efficacious than other forms of targeted MBI for individuals with ACE histories. Specifically, in helping to grow their sense of self and increase feeling of belonging. Moreover, such feelings of inclusion were evident alongside an increased sense of help-seeking. Again, demonstrating how the effects of MBIs for ACE experienced populations are interlinking and, more importantly, empowering to the individuals involved.

The current findings are particularly relevant due to ACE informed practice increasingly being at the forefront of policy making, e.g. Getting It Right For Every Child (GIRFEC) (The Scottish Government, 2012), Moreover, appropriate intervention is one of the key outcomes for The Mental Health Strategy 2017–2027 (The Scottish Government, 2017). By including samples from youth and adult age ranges this review provides preliminary support for the use of MBIs as both an early intervention, to foster positive changes for youth who experience adversity, and as a late intervention, for adults who have been surviving with the lasting impact of ACEs (Selous et al., 2019). Although further research into the magnitude of the effect of MBIs is needed to support this claim as well as systematic evaluation of potential harms, which we discuss further in the next section.

### Implications for Research

While the current findings have identified six themes to suggest that targeted MBIs may result in positive changes for ACE survivors, it was noted that little consideration is given to the potential negative outcomes that participants may experience. Indeed, only one study (Kimbrough et al., 2010) mentioned this, stating that no study-related effects were noted through their research process. This is unhelpful as it indicates that there may have been low level adverse effects but does not elaborate further. Worryingly, this study was the earliest published study included in the final review (Kimbrough et al., 2010), suggesting that a regression in the acknowledgement of adverse effects in the literature may have occurred. It should not be assumed from the omission of such information that adverse effects were not experienced, rather the likelihood is that they were not systematically evaluated. This is a common feature in psychological research generally with reviews finding consistently weak reporting of adverse effects (Duggan et al., 2014; Jonsson et al., 2014). One estimate suggests that only 28% of clinical research provides such data (Jonsson et al., 2014). Moreover, the current findings posit that underreporting in MBI research may be even lower, with only 8% of the included literature acknowledging adverse effects. This is particularly concerning because populations with ACE histories may be more susceptible to adverse effects of mindfulness than the general population, e.g. re-traumatization or deterioration in pre-existing clinical outcomes (Lindahl et al., 2017). The latter being very relevant due to the high levels of co-morbidity associated with ACEs (Felitti et al., 1998). It has been suggested that the reason for such underreporting of adverse effects is an effort to establish MBIs as evidence-based psychological interventions (Rozental et al., 2016). Regardless of rationale for doing so, a lack of reporting of adverse effects results in an inaccurate research record.

### Quality and Rigor of Reviewed Research

Methodological shortcomings were common, specifically, lack of random assignment, lack of follow-up, non-reporting of effect sizes, and lack of reporting of adverse events. Overall, the main methodological issues that arose during this review are not dissimilar to those apparent across intervention and efficacy research (Kazdin, 2015). By considering these shortcomings collectively, there appears to be more concern regarding the dissemination and application of MBIs being based on a lack of research, opposed to the quality of research in itself. This supports previous literature, which argues that the dissemination of MBIs may be ahead of its evidence base (Greenberg & Harris, 2012).

Future research could use this SMSR as a foundation upon which meta-analytical evaluation can be conducted. Finally, we recommend the adoption of open science and rigor and reproducibility methods including but not limited to pre-registration of study protocols (e.g., registered reports), the conduct of replication studies, secure and ethical data sharing, and carefully delineating both negative and positive effects of MBIs among people with ACEs when designing and reporting study data. A more rigorous, balanced, and open approach will lead to the strongest, most ethical and equitable scientific foundation from which interventions can be developed, deployed and evaluated.

### Strengths, Limitations, and Implications

The SMSR method is still relatively new compared to established review methods (Hong & Pluye, 2019; Saini & Shlonsky, 2012). However, it is methodologically inclusive, which allowed for the entire scope of the research pertaining to the research questions to be captured (Sandelowski et al., 2006, 2012). Moreover, the emotive impact of the improvements associated with the MBIs was apparent when extracting the data. This approach is relatively uncommon with most reviews that include mixed-methods data opting for a quantitative synthesis (Morse, 2012). Future research could replicate this SMSR method, adopting a quantitative approach and conduct a meta- analyses to further examine the magnitude of effect of MBIs on improving mental health and cognition among individuals with ACE histories.

## Supplementary Information

Below is the link to the electronic supplementary material.

Supplementary file1 (DOCX 27 KB)
