# Supplementary material for: The Efficacy of Targeted Mindfulness-Based Interventions for Improving Mental Health and Cognition Among Youth and Adults with ACE Histories: A Systematic Mixed Studies Review
Source: J Child Adolesc Trauma. 2022 May 5;15(4):1165–77. doi: 10.1007/s40653-022-00454-5 (PMC9684378; doi:10.1007/s40653-022-00454-5)
Supplement: Supplementary file 1 — Supplementary file1 (DOCX 27 KB) [file 40653_2022_454_MOESM1_ESM.docx]

| **Study ID** | **Quantitative Outcome Measures (when administered)** | **Qualitative Outcome Measures (when administered)** | **Mental health outcomes** | **Cognition outcomes** |
| --- | --- | --- | --- | --- |
| 1 | BDI, PCL, BSI, MAAS & home practice  Pre; 4/8/24 week follow up | Participant interviews 4 & 8 week follow up | BDI/depression score: baseline (22.1); 4 weeks post-intervention (13.7); 8 weeks post-intervention (7.8, *d*=1.8); 24 weeks  post-intervention (12.4, *d*=1.9). Improvement from baseline remained statistically significant at 24 week follow up, *F*=35.7, df=65, *p*<0.0001  BSI/anxiety score: baseline (1.7); 4 weeks post-intervention (1.0); 8 weeks post-intervention (0.9, *d*=01.1); 24 weeks post- intervention (1.0, *d*=0.9), *F*=15.0, df=65, *p*<0.0001  PCL/PTSD score: baseline (46.8); 4 weeks post-intervention (38.2); 8 weeks post-intervention (32.3, *d*=1.2); 24 weeks post- intervention (34.7, *d*=0.8). Improvement from baseline remained statistically significant at follow 24 week up, *F*=37.9, df=65, *p*<0.0001  MAAS/mindfulness score: baseline (3.0); 4 weeks post-intervention (3.5); 8 weeks post-intervention (4.0), *d*=1.2); 24 weeks post-intervention (3.8, *d*=1.0), *F*=15.0, df=65, *p*<0.0001 | ‡ |
| 2 | BDI & K- SADS  Pre; 2 weeks  post; 5 months follow up (Client A) & 3 months follow up (Client M) | ‡ | [Client A]  K-SADS/PTSD score: pre-intervention (2); post-intervention (1); 3 month follow up (0)  BDI/depression score: pre-intervention (20); session 5 (20); session 8 (8); session 12 (8); post-intervention (5); 3 month follow up (4)  [Client M]  K-SADS/PTSD score: pre-intervention (6); post-intervention (2); 3 month follow up (4)  BDI/depression score: pre-intervention (*not obtained*); session 3 (32); session 7 (25); post-intervention (13); 3 month follow  up (23) | ‡ |
| 3 | AAQ-II, WBSI, TSC-40 & VLQ  Pre; post; 8 month follow up | ‡ | TSC-40/trauma score: pre-intervention score (70); post-intervention score (45); 8 month follow up =(35) | AAQ-II/psychological inflexibility or experiential avoidance score: pre- intervention score (36); post- intervention score (26); 8 month follow up (26)  WBSI/thought suppression score: Pre-intervention (73); post-intervention score (57); 8 month follow up (35)  VLQ/valued living:  Stated values and behaviours pre- intervention score = (38); post- treatment (42), 8 month follow up (44).  Importance score: pre-intervention score (63); post-intervention score (66); 8 month follow up (64)  Consistency score: pre-intervention score (53); post-intervention score (56). 8 month follow up (61). |
| 4 | Wellness inventory; developed by author from the CSI, CSS and YSR)  Pre; post | ‡ | All results from the Wellness Inventory (developed by authors).  Pre/post-intervention differences for resilience (1.96 *p*<0.0019).  Boys resilience scores improved significantly (2.73, *p*<0.03). | Pre/post-intervention differences for social functioning (3.288, *p*=0.0014); behavior (0.006, *p*>0.05); cognitive functioning (0.510, *p*>0.05); emotional functioning (4.882, *p*<0.000).  Boys improved significantly on emotional functioning (7.03, *p*<0.000) & social functioning (6.29, *p*<0.002).  Girls had only one factor that achieved statistical significance: emotional functioning (3.17, *p*<0.025). |
| 5 | CTQ, ECR, CERQ, TMMS, RRQ, PANAS-X, DERS & FFMQ  Pre; post | ‡ | PANAS-X/negative emotion: non- significant differences in pre/post- intervention scores between groups, F=1.34, df=36. | Significant differences in pre/post- intervention scores between groups was found to be on:  RRQ/rumination, F=3.81, df=36, p<.05  CERQ/emotion suppression, F=2.70, df=36, *p*=.07  DERS/emotional dysregulation, F=4.71, df=36, *p*<.05  TMMS/emotional clarity, F=3.92, df=36, *p*<.05  FFMQ/mindfulness, F=12.30, df=36,  *p*<.01. |
| 6 | PSC-17, CAMM, STIAT/STAIS & ECG  Pre; post | Participant interviews & feedback/ interviews from observers Interviews at 9 weeks | Objective 1 [Intervention group]  PSC-17/general mental health (frequency, % of sample with positive scores):   - Externalised: pre-intervention = 4, 19.0%); post-intervention (3, 14.3%) - Internalised: pre-intervention = (4, 19.0%); post-intervention (6, 28.6%)   [Control group]  PSC-17/general mental health (% of sample with positive scores):   - Externalised: pre-intervention = (3, 14.3%); post-intervention (3, 14.3%) - Internalised: pre-intervention = (8, 38.1%); post-intervention (11, 52.4%)   Objective 3 [Intervention group]  CAMM/mindfulness: pre-intervention = (M=55.6, SD=8.3); post-intervention (M=56.0, SD=8.6)  STAIT/trait anxiety: pre-intervention = (M=42.6, SD=11.6); post-intervention (M=39.6, SD=8.8)  STAIS/state anxiety: pre-intervention = (M=40.8, SD=10.9); post-intervention (M=37.7, SD=8.6)  [Control group]  CAMM/mindfulness: pre-intervention = (M=12.6, SD=12.6); post-intervention (M=55.4, SD=11.3)  STAIT/trait anxiety: pre-intervention = (M=13.4, SD=13.4); post-intervention (M=42.5, SD=12.2)  STAIS/state anxiety: pre-intervention = (M=34.3, SD=11.1); post-intervention (M=40.2, SD=13.5) | Objective 1 [Intervention group]  PSC-17/general mental health (frequency, % of sample with positive scores):  Attention: pre-intervention = (3, 14.3%); post-intervention (3, 14.3%)  [Control group]  PSC-17/general mental health (% of sample with positive scores):  Attention: pre-intervention = (2, 9.5%); post-intervention (4, 19.0%) |
| 7 | CPSS, YOQ- SR, WAI-S, affirmation questionnaire & therapeutic factors inventory cohesiveness scale  Pre; post *(WAI-S post-intervention only)* | Yoga experiences questionnaire & individual interviews During (yoga experiences form); follow-up (individual interviews) | [Site 1]  YOQ-SR/overall mental health results found significant differences pre/post- intervention, t(20)=2.51, *p*=.021, *d* =.58 (moderate effect size)  CPSS/PTSD symptoms results found significant differences for pre/post- intervention scores, t(5)=2.51, *p*=.318, *d*=1.45 (large effect size)  [Site 2]  YOQ-SR/overall mental health results found no significant difference between pre/post intervention scores, t(6)=1.09, *p*=.318, *d* =.41 (small effect size)  CPSS/PTSD symptoms results found no significant between pre/post-intervention scores, t(6)=1.09, *p*=3.17, *d*=.30 (small effect size) | [Overall]  Average increase in calmness (M=3.04, SD=1.53) out of 10.  [Overall]  Affirmation Questionnaire results found significant differences pre/post intervention, t(29)=2.86, *p*=.008. |
| 8 | BPRS & GAD  Pre; post; 3 month follow up | ‡ | Significant differences (p<.05) were found between groups between baseline and post-intervention, and baseline and 3 month follow up on:  BPRS/psychiatric symptoms: β=-3.6, t(- 2.6), df=83.2*, p*>.5, *d*=.39  GAD/anxiety: β=1-1.8, t(-3.6), df=140.8, *p*>.5, *d*=.60. | Significant differences (p<.05) were found between groups between baseline and post-intervention, and baseline and 3 month follow up):  Help seeking, β=-0.8, t(-.28), df=119.2, *p*>.5, *d*=.43  Acceptance, β=1.05, t(2.0), df=139.6, *p*>.5, *d*=.37 |
| 9 | Mindfulness review survey  Post | Individual interviews & group interviews Pre; during; post | Mindfulness use per participant:  3 = frequent use (I.e. n=2 at >5, and n=1 at >3 times per week)  3 = high (once daily)  2 = low end (when needed).  *N.b. These results were used to guide qualitative outcomes and then embedded for final results.* | ‡ |
| 10 | ASQ, BPRS, GAD, SES, CTQ-SF & TMS  Pre; post; 3 month follow up | ‡ | Clinical and statistical difference between cluster change groups for:  BPRS/psychiatric symptoms, F(2,27)=14.9, *p*=0.00.  GAD/anxiety, F(2,27)=5.4, *p*=0.01. | Clinical and statistical difference between cluster change groups for:  CERQ/acceptance, F(2,27)=54.9,  *p*=0.00.  SES/help seeking, F (2,27)=4.6,  *p*=0.02. |
| 11 | I-PANAS-SF, DASS-21-D, CRIES  Pre; post | Semi- structured individual interviews Pre; post | Analysis (t-Test) found an overall significant decrease in negative affect, t=2.49, *p*=0.04, *g*=0.79, post-intervention.  An overall decrease, not significant, in depression symptoms, t=2.07, *p*=0.07, *g*=0.78; an overall increase in positive affect, again not significantly, after the intervention, t=-2.12, *p*=0.07, *g*=0.71). | ‡ |
| 12 | ‡ | Individual semi- structured interviews Pre; post Observations During |  |  |
| 13 | Primary outcomes: LSCL-SF & SBC  Secondary outcomes: PCL, SDQ, DES, BDI-II, BAI, PHLMS, IIP & SRS  Pre; post; 6 month follow up |  | Significant differences between intervention and control group for:  BAI/anxiety (F=6.30, *p*=0.02, *d*=.81) soothing receptivity scales (F=12.62, *p*=.001, *d*=1.12).  No overall effect on PCL /PTSD symptom scores (F=.14, *p*=.71, *d*=.12) - all participants had met criteria for PTSD using cut off score of 35 on PCL at baseline.  Notable, not significant, differences found for:  BDI/depression (F=3.32, *p=.08, d*=.59)  PHLMS/acceptance (F=3.85, *p*=.06, *d*=.61) | Significant difference between intervention and control group for body awareness (subscale of SBC) (F=8.15, *p*=.007, *d*=.91). |

‡ denotes no relevant data

# *Abbreviations*: A – adult age cohort (i.e. <19y); AAQ-II – Acceptance and Action Questionnaire-II; ACT – Acceptance and Commitment Therapy; ASQ – Ages and Stages Questionnaire; B – between age cohorts (i.e. 14-21y); BAI – Beck Anxiety Inventory; BDI – Becks Depression Inventory; BDI-II – Becks Depression Inventory-II; BPRS – Brief Psychiatric Rating Scale; BSI – Brief Symptom Inventory; C/A - child/adolescent age cohort (i.e. 18y); CAMM – Child Adolescent Mindfulness Measure; CERQ – Child Emotion Regulation Questionnaire; CPSS – Child PTSD Symptom Scale; CRIES – Children’s Revised Impact of Events Scale; CSI – Child Symptom Inventory; CSS – Child Strength Survey; CTQ – Childhood Trauma Questionnaire; CTQ-SF – Childhood Trauma Questionnaire- Short Form ; DASS-21-D – Depression, Anxiety and Stress Scale; DERS – Difficulties in Emotion Regulation Scale; DES – Dissociative Experiences Questionnaire; ECG – Electrocardiogram; ECR – Experiences in Close Relationships; F – female; FFMQ – Five Facet Mindfulness Questionnaire; GAD – General Anxiety Disorder Assessment; HCSAY – Healing Childhood Sexual Abuse with Yoga; I-PANAS-SF – International-Positive Affect Negative Affect Schedule-Short Form; IIP – Inventory of Interpersonal Problems; K-SADS – Kiddie-Schedule for Affective Disorders and Schizophrenia; LSCL-SF – Life Stressor Checklist-Short Form; M – male; MAAS – Mindfulness Attention and Awareness Scale; MBCT – Mindfulness-Based Cognitive Therapy; MBSR – Mindfulness-Based Stress Reduction; N – no; PANAS-X – Positive Affect Negative Affect Schedule; PCL – PTSD Checklist; PHLMS – Philadelphia Mindfulness Scale; PSC- 17 – Pediatric Symptoms Checklist; REAC2H - Restoring Embodies Awareness Compassionate Connection and Hope; RRQ – Rumination Reflection Questionnaire; SBC – Scale of Body Connection; SDQ – Strengths and Difficulties Questionnaire; SES – Service Engagement Scale; SRS – Social Responsiveness Scale; STAIT/STAIS – State Trait Anxiety Scale (T: trait/S: state) for Children; TBG – Trauma and the Body Group; TIY –Trauma Informed Yoga; TMMS – Trait Meta-Mood Scale; TMS – Toronto Mindfulness Scale; TSC-40 – Trauma Symptom Checklist-40; VLQ – Valued Living Questionnaire; WAIS –Wechsler Adult Intelligence Scale; WBSI – White Bear Suppression Inventory; Y – yes; YOQ-SR – Youth Outcome Questionnaire-Self Report; YSR – Achenbach Youth Self
